# Raman spectroscopy and artificial intelligence to predict the Bayesian probability of breast cancer

**DOI:** 10.1038/s41598-021-85758-6

**Published:** 2021-03-22

**Authors:** Ragini Kothari, Veronica Jones, Dominique Mena, Viviana Bermúdez Reyes, Youkang Shon, Jennifer P. Smith, Daniel Schmolze, Philip D. Cha, Lily Lai, Yuman Fong, Michael C. Storrie-Lombardi

**Affiliations:** 1 Department of Surgery, City of Hope National Medical Center, 1500 E. Duarte Rd, Furth 1116, Duarte, CA 91010 USA; 2 Department of Engineering, Harvey Mudd College, 301 Platt Blvd, Claremont, CA 91711 USA; 3 Department of Physics, Harvey Mudd College, 301 Platt Blvd, Claremont, CA 91711 USA; 4 Department of Pathology, City of Hope, 1500 E. Duarte Rd, Duarte, CA 91010 USA; 5 Kinohi Institute, Inc, Santa Barbara, CA 93109 USA

**Keywords:** Biophysics, Cancer, Breast cancer

## Abstract

This study addresses the core issue facing a surgical team during breast cancer surgery: quantitative prediction of tumor likelihood including estimates of prediction error. We have previously reported that a molecular probe, Laser Raman spectroscopy (LRS), can distinguish healthy and tumor tissue. We now report that combining LRS with two machine learning algorithms, unsupervised k-means and stochastic nonlinear neural networks (NN), provides rapid, quantitative, probabilistic tumor assessment with real-time error analysis. NNs were first trained on Raman spectra using human expert histopathology diagnostics as gold standard (74 spectra, 5 patients). K-means predictions using spectral data when compared to histopathology produced clustering models with 93.2–94.6% accuracy, 89.8–91.8% sensitivity, and 100% specificity. NNs trained on k-means predictions generated probabilities of correctness for the autonomous classification. Finally, the autonomous system characterized an extended dataset (203 spectra, 8 patients). Our results show that an increase in DNA|RNA signal intensity in the fingerprint region (600–1800 cm^−1^) and global loss of high wavenumber signal (2800–3200 cm^−1^) are particularly sensitive LRS warning signs of tumor. The stochastic nature of NNs made it possible to rapidly generate multiple models of target tissue classification and calculate the inherent error in the probabilistic estimates for each target.

## Introduction

Breast cancer is the most common cancer affecting women across the globe^1^. With the advancement of screening techniques, more breast cancers are caught at early stages^2^. Early stage breast cancer treatment includes breast conserving surgery and obtaining negative margins is paramount in preventing recurrence. Unfortunately, one in five patients will require re-excision surgery in order to achieve negative surgical margins^3^. As a result, the accurate determination of tumor margins in real time during surgical intervention has received significant attention^4,5^. Advanced technologies proposed to solve what has become known as “the margins problem” have included hyperspectral optical imaging^6^, magnetic resonance imaging^7^, and ultrasound^8^.

Amongst these technologies, Laser Raman Spectroscopy (LRS) is an emerging optical technique of considerable utility in surgical diagnostics. LRS probes the vibrational frequencies of molecular bonds to generate a unique biochemical signature for target tissues. The technique has been employed in diagnostic efforts for liver^9,10^, oral^11–15^, and prostate cancer^16–19^, as well as leukemia^20,21^, inflammation^22,23^, and apoptosis^24,25^. In breast cancer diagnostics, LRS can characterize microcalcifications^26–30^, distinguish immortalized, transformed, and invasive breast cancer cells^31^, and map the spatial distribution of carotenoids, mammaglobin, palmitic acid and sphingomyelin in ductal breast cancer^32^.

In brief, the excitement about LRS for breast cancer diagnosis is a response to the spectral specificity of the Raman scattering event, making it possible to quickly distinguish between lipid, protein, and DNA|RNA cell components^33,34^. LRS harnesses the vibrational frequencies of molecular bonds to provide a unique biochemical signature for target tissue. As a result, the technique can detect cellular changes characteristic of cancer tissue in vivo during the surgical procedure, facilitating real time margin evaluation. Here the morphological characteristics of breast cancer that should produce alterations in the LRS signal are quite clear: a massive increase in nuclear material and loss of cytoplasmic volume (predominantly lipids) compared to the healthy state^35,36^.

Currently, breast margin evaluation is most commonly performed by the pathologist following formalin fixation, paraffin embedding, thin sectioning, slide mounting, and staining of the tissue with haemotoxylin and eosin (H&E) stains. This process takes at least a day, and often longer. Haemotoxylin (purple) binds to acidic moieties such as DNA and RNA. Eosin (pink) binds to basic molecules. During slide preparation, cytoplasmic lipids are removed leaving behind structural proteins as spatial proxies. Most of these proteins are basic, including cytoplasmic filaments, intracellular membranes, and extracellular fibers. The classical H&E strategy not only requires binding pigments post-operatively, but cannot directly interrogate the lipid component of healthy or cancerous tissue. LRS can supply a similar cellular analysis in real time, as spectral signatures serve as proxies for the morphological alterations documented by histology.

LRS directly probes all the major cellular components without preparation: DNA, RNA, proteins, carbohydrates, and lipids. The spectra generated are so information-rich it has become quite common when employing LRS for surgical diagnostics to evaluate the entire Raman spectrum using principal component analysis (PCA) for initial feature extraction and data compression^37–42^. In a previous communication, we have presented data confirming that Raman spectral analysis using PCA in combination with linear discriminant analysis (LDA) can distinguish cancerous from healthy breast tissue using 16 bands gathered from the “fingerprint” region (here defined as 600–1800 cm^−1^) and 3 bands from the “high wavenumber” region (here defined as 2800–3000 cm^−1^)^43^.

However, LDA provided probabilistic estimates of tumor which were either quite high or quite low. For instance, running PCA and LDA on this current full dataset (n = 203) yielded high probabilities with an average of 1.0 and a standard deviation of 2.8e−04 and low probabilities with an average of 4.34e−03 and a standard deviation of 2.58e−02. Visual inspection of tumor and healthy spectra revealed that quite dissimilar spectra often received equally “certain” classification predictions from these algorithms. Hence in this paper we report on the use of stochastic neural networks (NNs) for transparent, statistically rigorous, probabilistic classification of healthy and tumor tissue. Additionally, we have noted that lipid components generate a significantly stronger Raman signal than both protein and DNA|RNA targets. Analyzing our own data and the published work of others, it appears that many of the spectral shifts reported as diagnostic for breast cancer may be due to the loss of lipid signals rather than detection of pathognomonic shifts in RNA, DNA, and protein composition. To evaluate this hypothesis, we have now investigated the ability of NNs to estimate the Bayesian probability that a Raman spectrum contains signatures characteristic of cancer using data from (1) the entire spectral bandwidth (600–3000 cm^−1^), (2) the fingerprint region (600–1800 cm^−1^), and (3) the high wavenumber region (2800–3000 cm^−1^).

In this communication, we first describe the information content of infrared Raman spectra characterizing healthy and cancer-containing breast tissue. We identify nine spectral regions useful in comparing DNA|RNA, protein, carbohydrate, and lipid cellular components of healthy and cancer cells. Six of these spectral regions originate in the fingerprint region (FP) (600–1800 cm^−1^) and three are collected in the high wavenumber (HW) region (2800–3000 cm^−1^). We first demonstrate the use of an unsupervised clustering algorithm, k-means, to initially identify clusters of healthy and cancerous targets. We compare the spectral data partitioning to the human expert classification using standard clinical histopathology. We then present the results of training three NNs to estimate the Bayesian probability that a target exhibits the LRS signatures expected from cancer tissue. One NN, FPHW, provides a broadband analysis of the spectral data using all nine bands. The two other networks focus on data from just the FP (6 bands) or HW (3 bands) regions. We demonstrate that the inherent stochastic nature of NNs make it possible to rapidly generate multiple sets of target tissue classification and then use those analyses to calculate the inherent error in the probabilistic estimates for each target. Our data indicate that loss of signal in HW bands may serve as an early warning marker of tissue destruction, while several FP bands may be particularly sensitive to subtle shifts in RNA, DNA, and protein composition. Finally, we illustrate the use of stochastic NNs to evaluate the unsupervised k-means classification of 203 spectra from 8 patients with ductal breast cancer independent of histopathological diagnostics.

## Results

The pathologist on our team (DS) estimated the amount of cancer present in each Raman spectral target area using a semi-quantitative five-point scale ranging from 0 (no evidence of tumor) to 100% (all regions in the target area involved to some extent by tumor tissue). We collected a total of 203 samples out of which 154 were correlated with an H&E image and labelled with a quintile assessment of tumor involvement. See “Methods” for full description of slide preparation and imaging.

**Figure 1:**
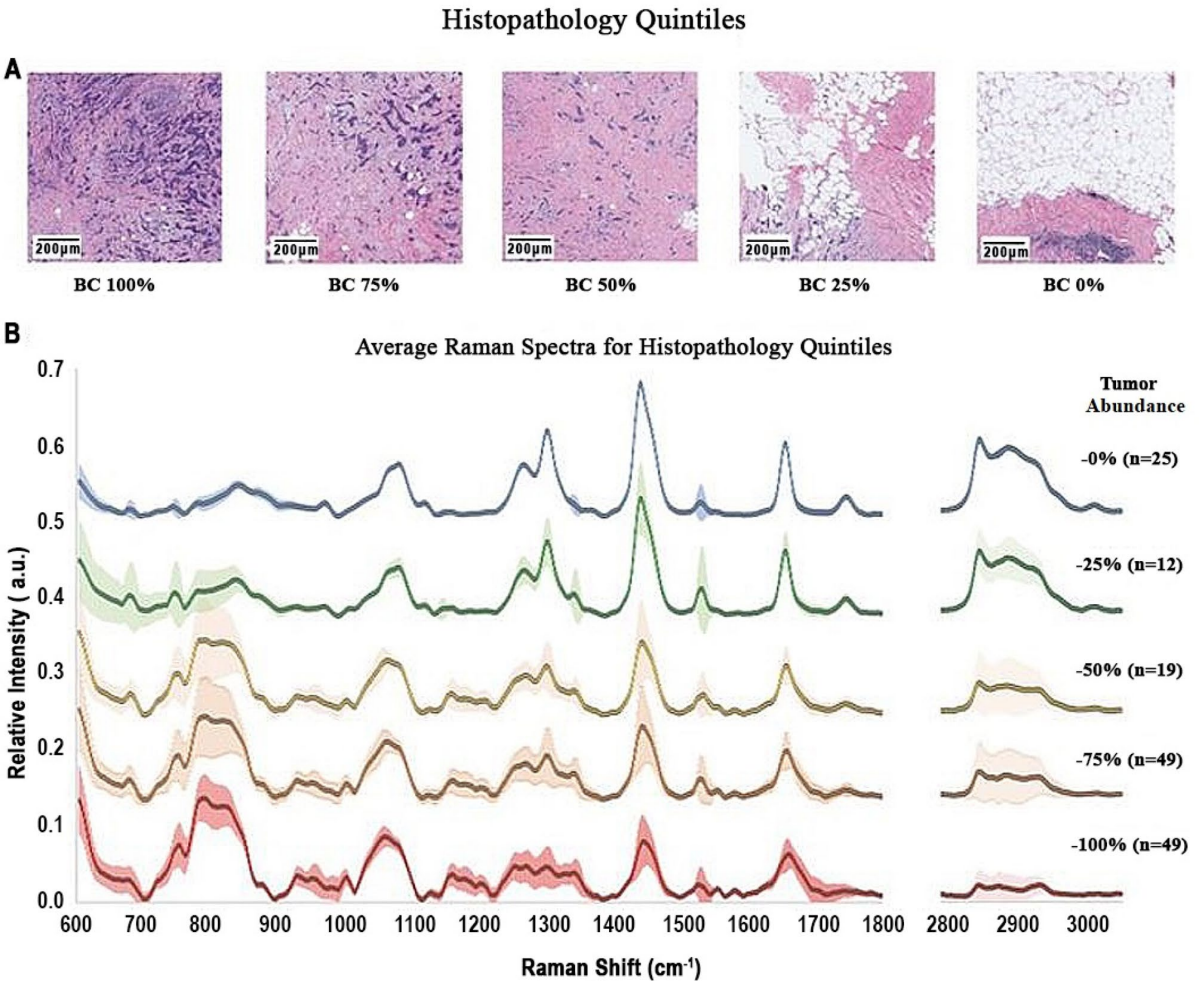
Breast cancer histopathology and corresponding laser Raman spectra (n = 154, 5 patients). Quintile estimate were made by the clinical pathologist (DS) for 1 mm^2^ areas surrounding the projected location of the Raman laser probe. (**A**) Shows the H&E stains for examples of the five tissue categories. Mean LRS spectrum for each of the five categories (n0 = 25; n25 = 12; n50 = 19; n75 = 49; n100 = 49) appear in (**B**). If a region is assigned to the 100% quintile it means that tumor cells are apparent in all portions of the image. If a region is assigned to the 0% quintile it means that there were no clusters of tumor cells apparent throughout the image. Quintile assignment was accomplished for 154 images and spectra acquired from five (5) patients. Average spectra are displayed along with 1-sigma error bars. Scale bars are 200 µm.

Figure 1A shows the H&E stains for examples of the five of the tissue categories. These are 1 mm^2^ regions surrounding the target site for the Raman data acquisition. Healthy tissue appears red where eosin dye has bound to cell structural proteins. Healthy regions also contain empty spaces where paraffin has replaced lipids during slide preparation. Tumor-rich tissue appears blue and purple where hematoxylin dye has bound to DNA, RNA, and peri-nuclear proteins. Figure 1B shows the mean LRS spectrum for each of the five regions along with 1-sigma error bars (n_0_ = 25; n_25_ = 12; n_50_ = 19; n_75_ = 49; n_100_ = 49).

**Figure 2:**
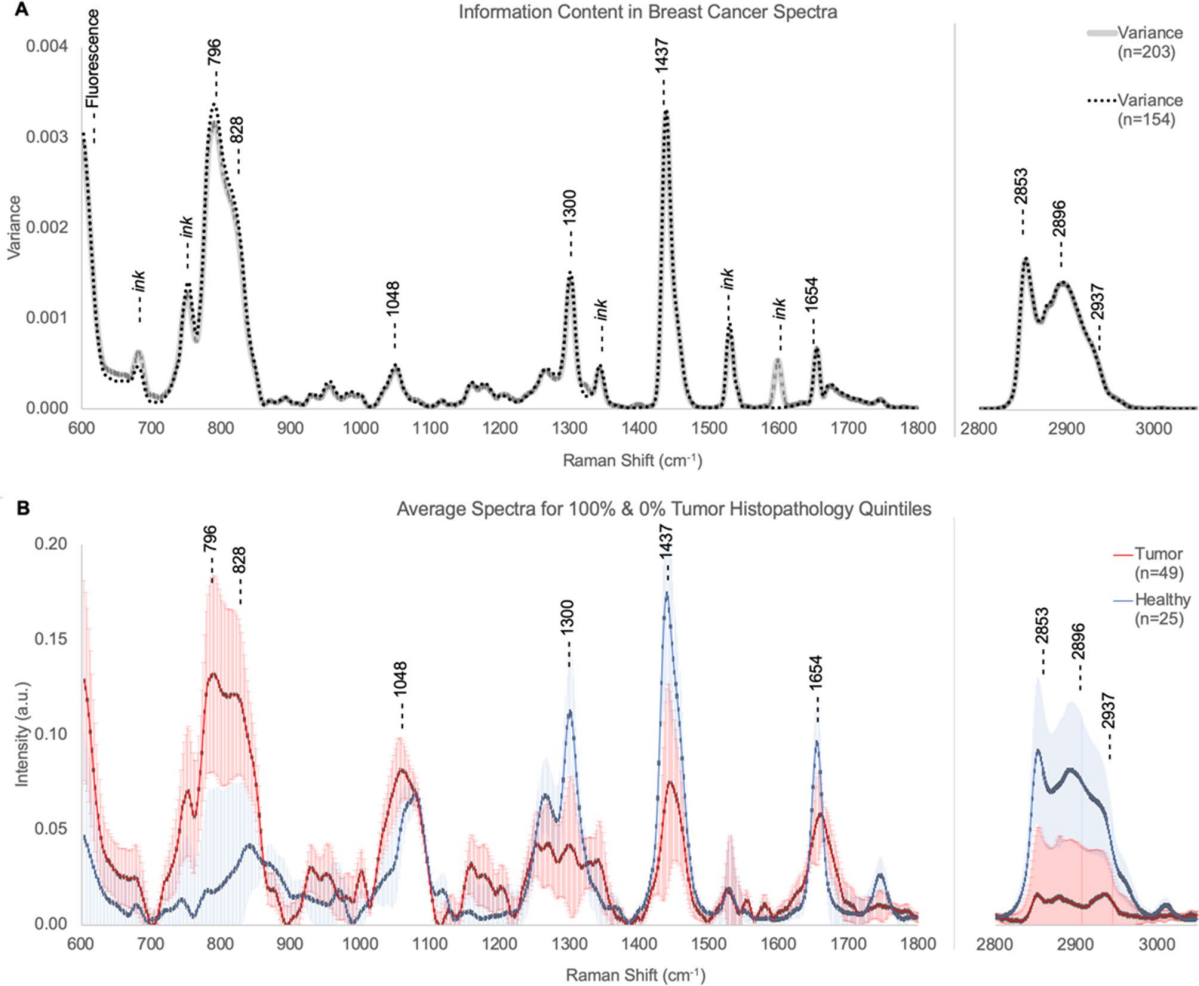
Information content of laser Raman spectra of healthy and cancerous tissue. Spectral variance for the full data set (203, 8 patients) and for the histopathology subset (n = 154, 5 patients) appears in (**A**). Spectral regions known to be at risk for contamination by surgical ink are noted. The 9 bands selected from the most information-rich (high variance) regions without evidence of ink contamination are shown. See Fig. S2 in Supplementary materials for Raman spectra of surgical inks. See Table 1 for band assignments. (**B**) Shows the average spectra with 1-sigma error bars for the histopathology 100% (Tumor, n = 49) and 0% (Healthy, n = 25) quintiles.

Since Raman spectra are high dimensional, feature extraction is necessary to avoid overfitting during neural network training. This is achieved by selecting regions with the highest variance (most information) across both the entire dataset (n = 203, 8 patients) and the subset of spectra assigned to histopathology quintiles (n = 154, 5 patients). Figure 2A shows the variance for both the entire dataset and the histopathology subset with the nine bands chosen as features for the classification algorithms. The peak intensity for the bands and their probable biochemical origin appear in Table 1. Figure 2B provides a direct comparison of the mean ‘healthy’ (0%, n_0_ = 25) and ‘tumor’ (100%, n_100_ = 49) spectra along with 1-sigma error bars. For a comparison between this heuristic feature selection and PCA see Supplementary material.

**Table 1:** Assignment of Raman spectral bands.

Peak position (cm^−1^)	Assignment
796	DNA|RNA ring breathing modes and O–P–O backbone^33,34,63–69^
828	Proline, hydroxyproline, tyrosine, O–P–O)^68,70,71^; mono- and polysaccharides^63,72,73^
1048	Symmetric stretch vibration of ν_3_PO_4_^3-^ in hydroxyapatite^70^ and glycogen^67^
1300	Lipids^63,69–72,74–78^, collagen^70,79^, protein amide III^68^
1437	Lipids^14,63,71,77^, fatty acids^73–75^, triglycerides^80^, collagen^70,78,79,81^, and phospholipids^78,81^
1654	Amide I if collagen assignment, and/or C=C of lipids in normal tissue^37,75^
2853	Symmetric stretch of lipids^78,82^
2896	Asymmetric stretch of protein, lipids, glycogen^71,82–84^
2937	C–H vibrations in lipids, proteins, glycogen, DNA|RNA^71,84,85^

Nine Raman bands were identified known to provide information on multiple cellular components including DNA|RNA, proteins, carbohydrates, and lipids (Table 1).

The bands are used as inputs to simple 3-layer neural networks with configuration 9:3.2; 6:3.2; or 3:3.2, i.e. 9, 6, or 3 input nodes, 3 hidden nodes, and 2 output nodes. Specifically, for NN FPHW (9:3.2), Raman fluxes from 6 bands in the fingerprint region (FP) were combined with three bands from the high wavenumber (HW) region to serve as 9 inputs for a network. NN FP (6:3.2) used the six bands from the finger print region, and NN HW (3:3.2) employed as inputs the three bands found in the high wavenumber region. Like the full spectrum data set used for FPHW, these inputs provided a mixture of information on all four of the primary cellular constituents: DNA|RNA, proteins, carbohydrates, and lipids.

First, k-means was run on the eigenvector dataset (0%, n = 25 and 100%, n = 49 tumor), in order to compare it to the human expert histopathology classification. Table 2 shows the number of spectra in the each of the two clusters (healthy, tumor) generated by k-means.

**Table 2:** Number of spectra in cluster 1 (healthy) and cluster 2 (tumor) generated by k-means unsupervised clustering.

Dataset	Cluster 1 (healthy)	Cluster 2 (tumor)
FPHW	29	45
FP	30	44
HW	29	45

Table 3 shows the prediction statistics for the k-means classification of the targets with histopathology classifications of 100% (n = 49) and 0% (n = 25) likelihood of tumor. These prediction statistics simply represent the k-means clustering of spectral data compared to the histopathology quintile classification of 100% or 0% tumor (N = 74). Accuracy was 94.5% for FPHW and HW, and 93.2% for FP. Sensitivity was 91.8% for both FPHW and HW, and 89.8% for FP. Specificity was 100% for all three.

**Table 3:** Prediction statistics for comparison between histopathology gold standard for 0% (n = 25) and 100% (n = 49) quintile categories and the unsupervised k-means prediction (k = 2) for FP, FPHW and HW datasets.

K-means versus histopathology prediction statistics			
Dataset	Accuracy (%)	Sensitivity (%)	Specificity (%)
FPHW	94.59	91.84	100
FP	93.24	89.80	100
HW	94.59	91.84	100

**Figure 3:**
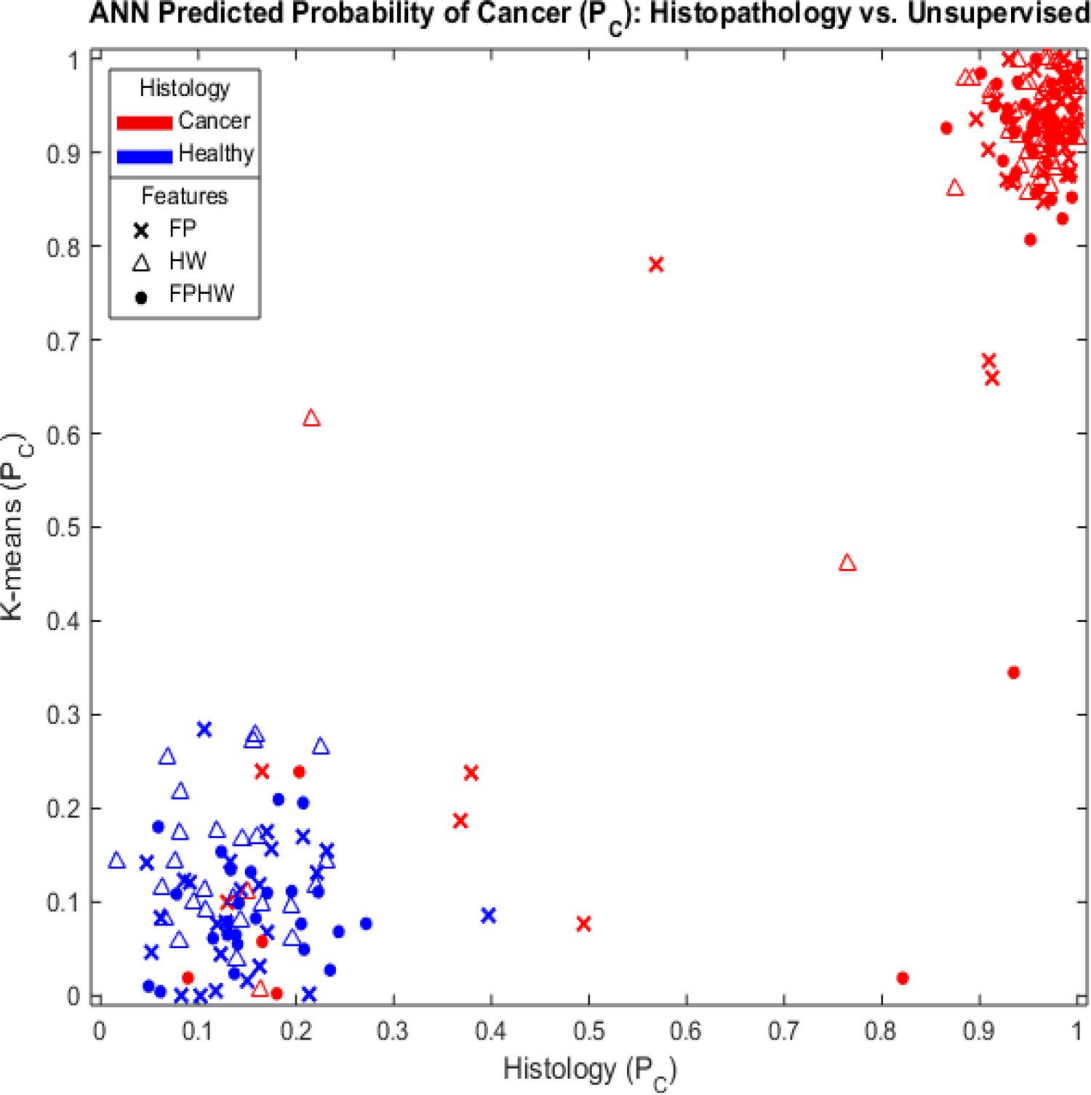
Bayesian probabilistic classification for 74 spectra from 5 patients using either H&E staining (x-axis) or k-means unsupervised spectral clustering (y-axis) as training gold standard. Spectra were from regions classified by histopathology as likely to be composed of either 100% or 0% tumor. Networks were trained using as inputs (x) 6 bands from the FP region (FP); (△) 3 bands from the HW region (HW); or (●) the full set of 9 bands (FPHW). Targets obtained from tissue in tumor-rich regions according to H&E stain are denoted by red markers, while data obtained from areas apparently devoid of tumor are denoted by blue markers.

Tumor probabilities produced by the NNs trained using k-means spectral classification were compared to the predictions by NNs trained using histopathology classification as the gold standard. The results appear in Fig. 3. Spectra were obtained from regions classified by histopathology as likely to be composed of either 100% tumor or 0% tumor. Networks were trained using as inputs (x) 6 bands from the FP region (FP); (△) 3 bands from the HW region (HW); or (●) the full set of 9 bands (FPHW). Targets obtained from tissue in tumor-rich region according to H&E stain are denoted by red markers, while data obtained from areas apparently devoid of tumor are denoted by blue markers. This analysis is repeated with balanced classes (Healthy, Tumor, n = 25) in Supplementary materials (See Supplementary Fig. S1).

The k-means clustering and human expert histopathology classification of 100% and 0% tumor regions disagreed on five (5) spectra. Table 4 shows the NN (trained on k-means) generated probabilities for these 5 spectra for all three datasets (FP, HW, FPHW) as well the average probability. Predictions of tumor likelihood > 20% appear in bold in the table.

**Table 4:** Neural network (trained on k-means) prediction of tumor likelihood for five spectra from regions are shown to be rich in tumor tissue by histopathology, but predicted to be “healthy” by k-means.

Neural network prediction of tumor likelihood					
NN	s1 (%)	s2 (%)	s3 (%)	s4 (%)	s5 (%)
FPHW	18.5	4.6	0.1	**23.8**	**61.2**
FP	4.2	8.6	10.1	11.1	13.4
HW	11.3	13.1	**20.4**	8.5	**82.0**
Average NN	11.4	8.7	10.2	14.5	**52.2**

**Figure 4:**
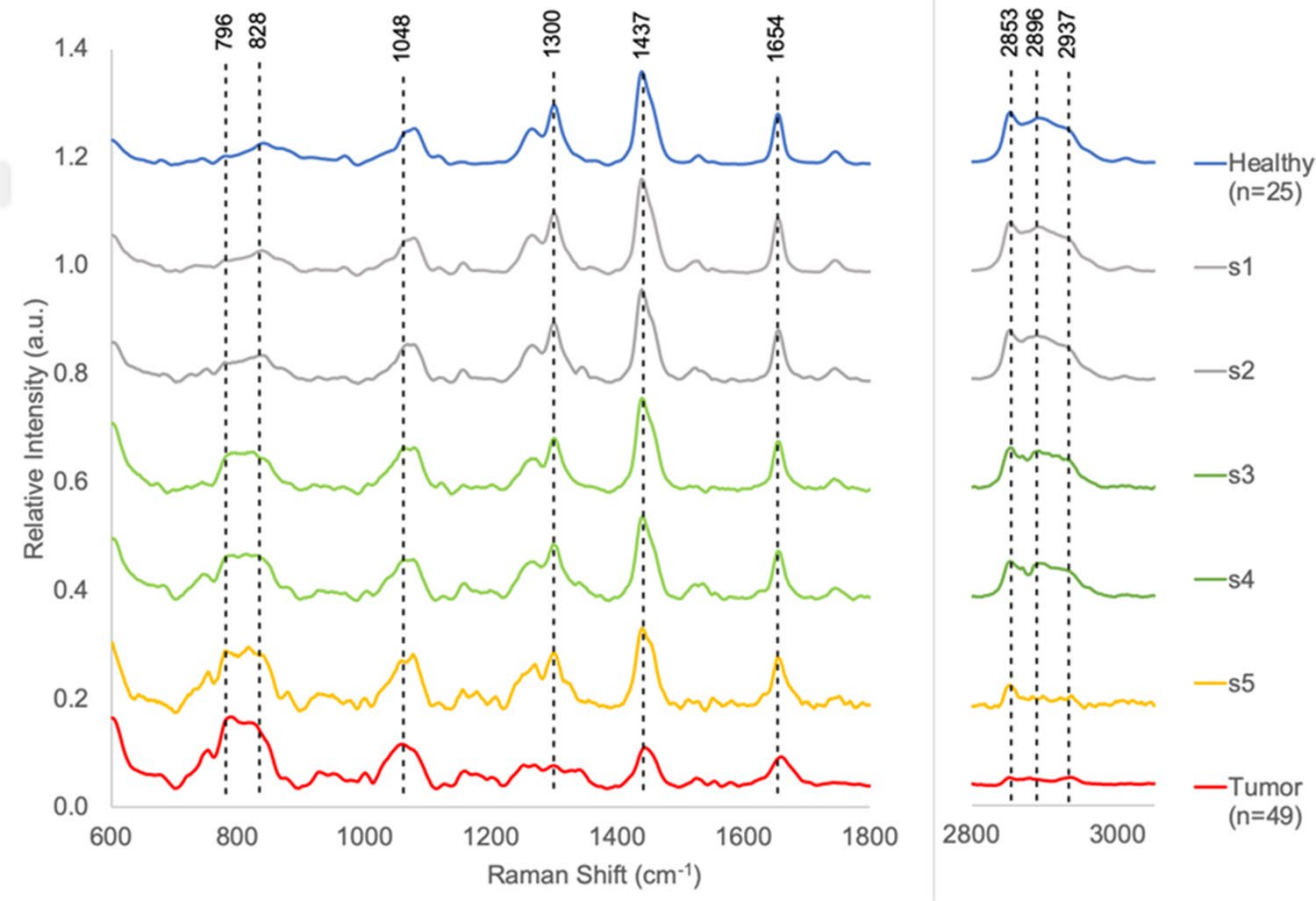
Disagreement between Histopathology and k-means. Spectra s1 to s4 were acquired form H&E regions assessed to be 100% tumor by histology, but were assessed by k-means to be most similar to healthy spectra. The results were the same for all 3 feature sets (FP, HW, and FPHW). Spectrum s5 was also obtained from a region assessed as 100% infiltrated with tumor tissue by H&E analysis. The spectrum was classified by k-means as cancerous when using HW and FPHW data sets, but was classified as healthy when using the FP feature set. Spectra s1 and s2 (gray) are most similar to the average healthy spectrum shown in Fig. 1 and reproduced here at the top of the graph (blue). The similarity is evident both to qualitative visual inspection and the two spectra appear at the heart of the k-means cluster for healthy spectra. Healthy features include the relatively weak RNA|DNA backbone and protein signal at 796 and 828 cm^−1^, respectively with and the strong signal in HW. Spectra s3 and s4 (green) show characteristics of both healthy and tumor spectra. The RNA|DNA backbone and protein signals are increasing with the signal at 796 cm^−1^ increasing relatively more than the 828 cm^−1^ band, but the strong signal in HW remains. Spectra s5 (gold) is most similar to the average cancer spectra. Here the HW signal as collapsed and nucleotide and protein signals have increased, but the band intensity ratio for 796/828 has not shifted to favor the nucleotide moiety as it has in the average tumor spectrum (red) at the bottom of the graph.

Likelihoods of tumor > 20% appear in bold. Figure 4 shows these 5 spectra along with average spectra from regions classified as 0% and 100% tumor.

Figure 4 shows these five spectra (labeled s1 to s5) along with the average spectra from the healthy and tumor regions (quintile 0% and 100%).

The five spectra were acquired from H&E regions assessed to be 100% tumor by histopathology. Spectra s1–s4 were predicted by unsupervised k-means to be most similar to healthy spectra using any one of the 3 feature sets (FP, HW, and FPHW). The s5 spectrum was classified by k-means as cancerous when using HW and FPHW data inputs, but as healthy when using the FP feature set. Spectra s1 and s2 (rendered in gray in Fig. 4) are most similar to the average healthy spectrum. The similarity is evident both to qualitative visual inspection and the two spectra appear near the center of the k-means cluster for healthy spectra. Healthy features include the relatively weak RNA|DNA backbone and protein signal at 796 and 828 cm^−1^, respectively, with the strong signal in HW. For spectra s1 and s2 all three NN configurations generate Bayesian estimates of tumor likelihood of < 20%. Spectra s3 and s4 (green) show characteristics of both healthy and tumor spectra. The RNA|DNA backbone and protein signals are increased with the band at 796 cm^−1^ increasing relatively more than the 828 cm^−1^ band, but the strong signal in HW so characteristic of healthy tissue remains relatively intact. Spectra s3 and s4 exhibit an increase in the RNA|DNA backbone and protein signature and a decrease in the high wavenumber signal expected for tumor. For spectrum s3 HW, the NN trained on high wavenumber data, generates a 20.4% likelihood of tumor. The NNs trained solely on fingerprint region data, FP, produces only a 10.1% likelihood of tumor. Similarly, for s4 FPHW produced a 23.8% likelihood of tumor. Spectra s5 (gold) is qualitatively most similar to the average cancer spectra. Here the HW signal has collapsed while the nucleotide and protein signals have increased further. In addition, the band intensity ratio for 796/828 has now shifted to favor the nucleotide moiety as it has in the average tumor spectrum (red) at the bottom of the graph. NNs HW and FPHW generate significant tumor likelihood for spectrum s5 (HW ~ 82.0%, FPHW ~ 61.2%).

Unsupervised k-means clustering and NN probability generation predict the likelihood of tumor for a larger dataset (n = 203, 8 patients) using all three feature sets (FP, HW, FPHW). The number of spectra in each k-means cluster for each of the three datasets appear in Table 5. All Raman spectra visually agreed with cluster assignment. These cluster assignments are further assessed through NN Bayesian probability estimation of how likely the k-means cluster assignment is correct. The use of stochastic NNs and three NN configurations makes it possible to generate two types of information (variance) to assess the reliability of the Bayesian probability of cancer. In this experiment, the k-means classification is employed as the gold standard for 10 train-test cycles with each of the three NN configurations. Two types of variance are measured. First, the variance in the output for each NN from the 10 test-train cycles is determined and designated the “intra-NN variance” or ***V***_***RA***_. The lower the *V*_*RA*_, the more certain the NN is of its prediction of tumor likelihood (*P*_*Tumor*_). The second variance, designated the inter-NN variance (***V***_***ER***_), is generated using the 10-run average output probabilities (*P*_*Tumor*_) of the 3 NN configurations. Increases in *V*_*ER*_ indicate disagreement amongst the three NN configurations in their estimate of *P*_*Tumor*_. These two types of variances represent the reliability and reproducibility of the final algorithm (k-means and neural network) for the full dataset (n = 203). Since leave-one-out cross validation was employed in the neural network analysis, these two variances are also our best safeguard for overfitting. The hallmark of a Bayesian estimator is that the probabilities of the two events sum to one. We have summed the probabilities for the two output classes of the NNs and confirmed that they sum to one as another safeguard for overfitting.

**Table 5:** Number of spectra in each class for k-means on full dataset (n = 203).

Dataset	Cluster 1 (healthy)	Cluster 2 (tumor)
FP	88	115
FPHW	86	117
HW	87	116

This remains consistent over multiple re-runs of the algorithm.

**Figure 5:**
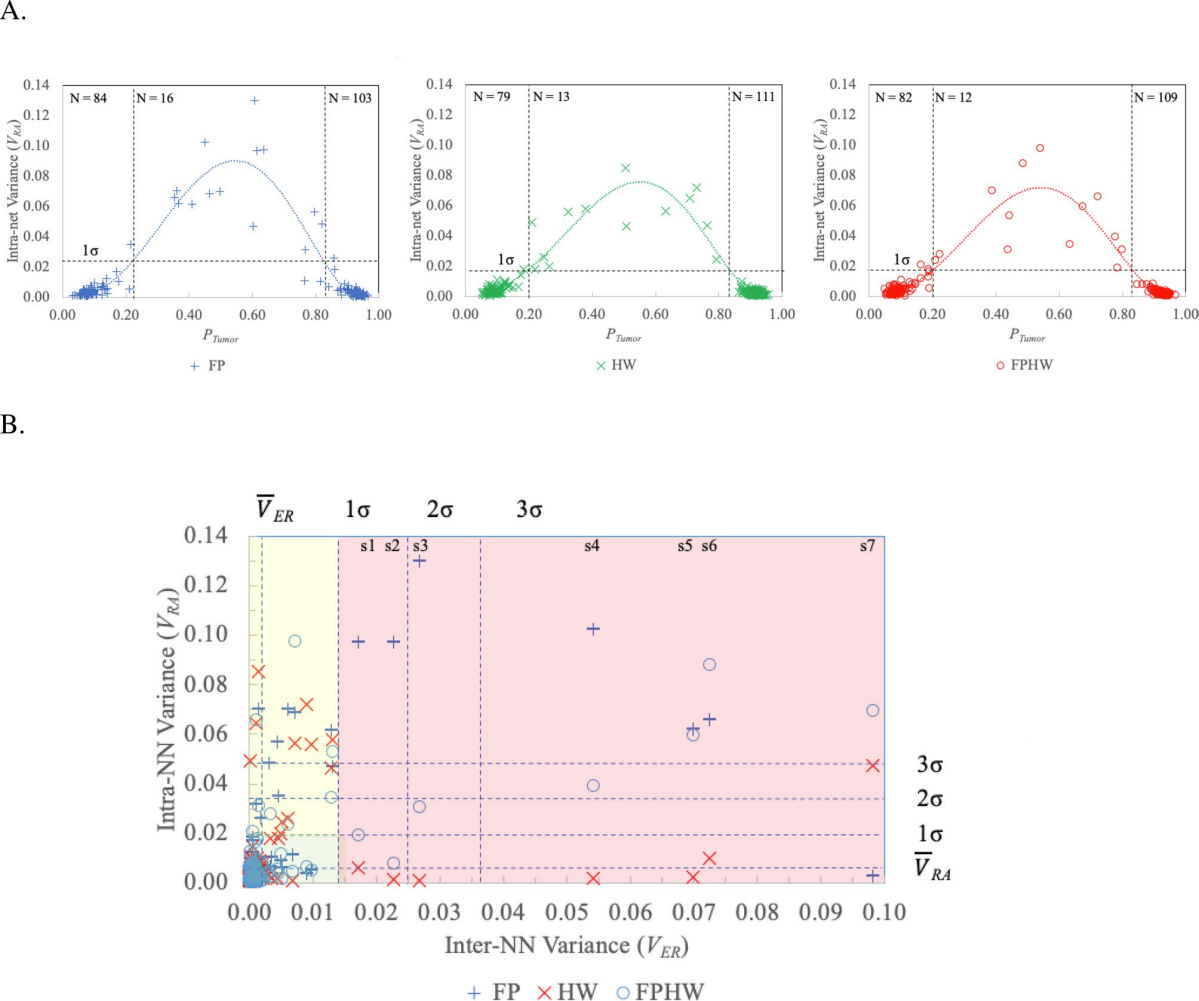
Characterizing the variation in the output of each stochastic NN (intra-NN variance or *V*_*RA*_) and the differences in predicted tumor likelihood amongst the NN configurations (inter-NN variance, *V*_*ER*_). The use of 3 configurations of stochastic NNs provides two useful variance estimates for assessing NN reliability and enhancing tumor detection likelihood estimates. The output variance of each stochastic NN when run through 10 complete train-test cycles with random weight resets for each cycle provides an intra-NN output variance estimate (*V*_*RA*_) for each of the three NN configurations. The lower the *V*_*RA*_, the more certain the NN is of its prediction. The inter-NN variance (*V*_*ER*_) is then calculated as the variance in the average output probabilities (*P*_*Tumor*_, N = 10) generated by the 3 NN configurations while determining the *V*_*RA*_ variance for each. *V*_*ER*_ provides an immediate detection of disagreements in diagnostic predictions for the three NN configurations. (**A**) Depicts *V*_*RA*_ for each of the three NN configurations as a function of *P*_*Tumor*_ between the NN training boundaries (*P*_*Tumor*_ ~ 0 =  > healthy, *P*_*Tumor*_ ~ 1 =  > tumor). *P*_*Tumor*_ estimates are divided into those with *V*_*RA*_ < 1σ and *V*_*RA*_ > 1σ. Vertical dotted lines indicate the point where a 5^th^ order polynomial fit to the data intersects at the 1σ level. Significant increases in *V*_*RA*_ (*V*_*RA*_ > 1 σ) appear as expected in the boundary zone between the two classes (i.e., ~ 0.2 < *P*_*Tumor*_ <  ~ 0.8). (**B**) Depicts *V*_*RA*_ as a function of *V*_*ER*_. Significant inter-NN variance (*V*_*ER*_ > 1σ) occurred in 7 targets (shaded red), all from tumor or boundary regions. In six of the seven high *V*_*ER*_ samples, the HW NN exhibits the least variance in its predictions (V_*RA*_ between 0.001 and 0.10). For the other target (s7) FP exhibits the minimal variance across ten trials (*V*_*RA*_ ~ 0.003). Spectra corresponding to these 7 targets appear in Fig. 6. See Table 6 for a comparison of the cancer probability predictions and the NN variances. Another 17 targets (yellow shading) exhibit high *V*_*RA*_ (> 1σ), but low *V*_*ER*_ (< 1σ). See Table S2 in Supplementary Material.

Figure 5A depicts *V*_*RA*_ for each of the three NN configurations as a function of *P*_*Tumor*_ between the NN training boundaries (*P*_*Tumor*_ ~ 0 =  > healthy, *P*_*Tumor*_ ~ 1 =  > tumor).

*P*_*Tumor*_ estimates are divided into those with *V*_*RA*_ < 1σ and *V*_*RA*_ > 1σ. Vertical dotted lines indicate the point where a 5th order polynomial fit to the data intersects at the 1σ level. Significant increases in *V*_*RA*_ (*V*_*RA*_ > 1 σ) appear as expected in the boundary zone between the two classes (i.e., ~ 0.2 < *P*_*Tumor*_ <  ~ 0.8). Figure 5B depicts *V*_*RA*_ as a function of *V*_*ER*_. Significant inter-NN variance (*V*_*ER*_ > 1σ) occurred in 7 targets (shaded red), all from tumor or boundary regions. Table 6 compares the cancer probability predictions (*P*_*Tumor*_) and the NN variances (*V*_*RA*_ and *V*_*ER*_) for the 7 high samples.

**Table 6:** Using variance in predicted tumor likelihood amongst the three NN configurations (inter-NN variance, *V*_*ER*_) to identify tumors predicted by at least one but not all NNs.

Figure 6	Histology^a^	Region^b^	NN tumor prediction			Inter-NN variance (*V*_*RA*_)	Intra-NN variance (*V*_*ER*_)		
			FP	HW	FPHW		FP	HW	FPHW
s1	100	B	0.615	**0.871**	0.785	0.017	0.097	**0.006**	0.019
s2	100	T	0.635	**0.927**	0.846	0.023	0.097	**0.002**	0.008
s3	–	T	0.606	**0.932**	0.800	0.027	0.13	**0.001**	0.031
s4	100	B	0.448	**0.897**	0.777	0.054	0.103	**0.002**	0.039
s5	100	B	0.366	**0.891**	0.674	0.07	0.062	**0.002**	0.06
s6	100	B	0.354	**0.873**	0.487	0.072	0.066	**0.010**	0.088
s7	75	T	0.141	**0.763**	0.387	0.098	**0.003**	0.047	0.07

Significant inter-NN variance (see Fig. 5B, *V*_*ER*_ > 1σ) occurred in 7 targets, all from tumor or boundary regions. The highest NN tumor probability for each target appears in bold. NNs using only FP inputs predicts 3 of these targets contain tumor, but with relatively low probabilities (*P*_*Tumor*_ = 0.62 ± 0.02, N = 3). FPHW using the full nine inputs predicts 5 of the 7 targets contain tumor and produces higher probabilities (*P*_*Tumor*_ = 0.78 ± 0.06, N = 5). The NNs trained only on HW data predict all 7 of these targets contain tumor. HW NNs generated the highest tumor likelihood probabilities (*P*_*Tumor*_ = 0.88 ± 0.06, N = 3). The variation in the output of each stochastic NN (train-test cycle repeated 10 times with random weight restarts) provides an intra-NN variance or ***V***_***RA***_ for each of the three NN configurations. Six of the seven HW NNs exhibits the least variance in their predictions (V_*RA*_ between 0.001 and 0.10). One a single target (s7) NN FP exhibits minimal variance across ten trials (*V*_*RA*_ ~ 0.003) while generating a low tumor probability (*P* ~ 0.141). For target s7 HW predicts a tumor likelihood of *P* ~ 0.763 with *V*_*RA*_ = 0.047.

**Figure 6:**
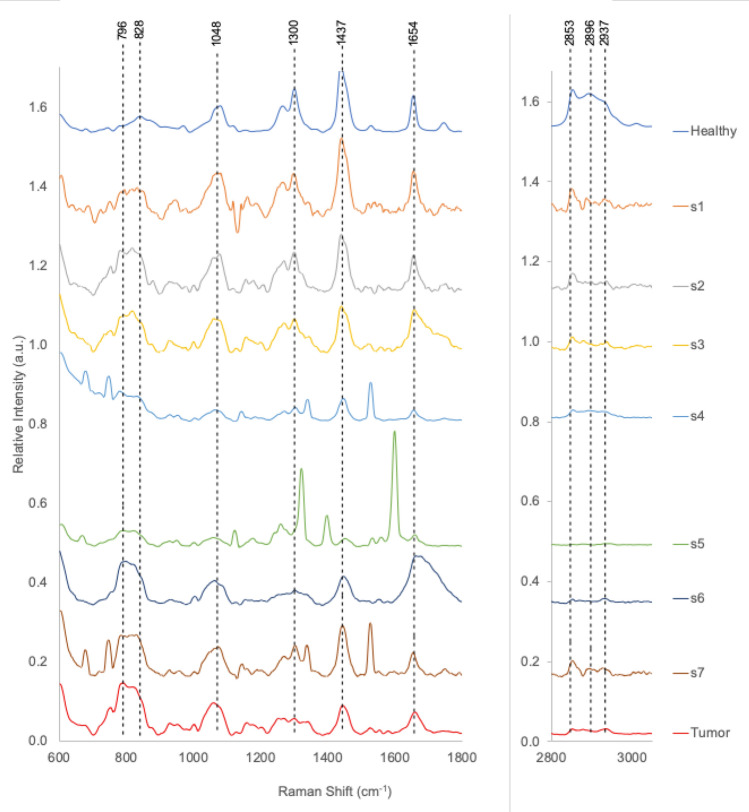
Spectra corresponding to the 7 high *V*_*ER*_ targets listed in Table 6. The average spectra from tumor and healthy regions appear at bottom and top of figure, for comparison. All of the 7 spectra show a depletion in signal strength for the three HW bands. The HW NN cancer likelihood probabilities ranged from *P*_*Tumor*_ ~ 0.763 to 0.927. s7 produces the lowest HW tumor probability (*P*_*Tumor*_ = 0.763) and highest *V*_*ER*_ (~ 0.047) due to the residual signal at 2853 cm^−1^. Spectra s5, s6, and s7 triggered a low-level warning in FP (*P*_*Tumor*_ ~ 0.6). These spectra show increases in signal strength at 796 and 828 cm^−1^, indicative of cellular increases in nucleic acids and proteins. Spectra s4, s5, and s7 contain narrow peaks of activity expected from surgical marking dyes at locations that do not compromise the nine NN diagnostic bands.

^a^Pathologist target labels in Fig. 6. Pathologist estimate of likelihood probe would strike tumor tissue in 1 mm^2^ region around laser.

^b^Macroscopic (1X) visual assessment of spectra collection sites as tumor (T), healthy (H), or boundary (B) regions.

The highest *P*_*Tumor*_ estimate and the lowest *V*_*RA*_ for each target appears in bold. These are data points worth investigating since there is high disagreement between the nets (high *V*_*ER*_,) but each net is confident in its’ output (low *V*_*RA*_). NNs using only FP inputs predict 3 of these targets contain tumor, but with relatively low probabilities (*P* = 0.62 ± 0.02, N = 3). FPHW using the full nine inputs predicts 5 of the 7 targets contain tumor and produces higher probabilities (*P* = 0.78 ± 0.06, N = 5). The NNs trained only on HW data predict all 7 of these targets contain tumor. HW NNs generated the highest tumor likelihood probabilities (*P*_*Tumor*_ = 0.88 ± 0.06). In six of the seven high *V*_*ER*_ samples, the HW NN exhibits the least variance in its predictions (V_*RA*_ between 0.001 and 0.10). For the remaining target (s7), FP exhibits the lowest variance across ten trials (*V*_*RA*_ ~ 0.003), but generated a tumor likelihood *P*_*Tumor*_ ~ 0.141. For that same target (s7) the HW NN predicted a tumor likelihood of P ~ 0.763 with *V*_*RA*_ = 0.047.

Spectra corresponding to these 7 targets appear in Fig. 6. The average spectra from tumor and healthy regions appear at bottom and top of figure, for comparison. All of the 7 spectra show a depletion in signal strength for the three HW bands. The HW NN cancer likelihood probabilities ranged from *P*_*Tumor*_ ~ 0.763 to 0.927. s7 produces the lowest HW tumor probability (*P*_*Tumor*_ = 0.763) and highest *V*_*ER*_ (~ 0.047) due to the residual signal at 2853 cm^-1^. Spectra s5, s6, and s7 triggered a low-level warning in FP (*P*_*Tumor*_ ~ 0.6). These spectra show increases in signal strength at 796 and 828 cm^−1^, indicative of cellular increases in nucleic acids and proteins. Spectra s4, s5, and s7 contain narrow peaks of activity expected from surgical marking dyes at locations that do not compromise the nine NN diagnostic bands.

Seventeen targets (Fig. 5B, yellow shading) exhibit high *V*_*RA*_ (> 1σ), but low *V*_*ER*_ (< 1σ). See Table S2in Supplementary material for *P*_*Tumor*_, *V*_*RA*_, and *V*_*ER*_ values. For these targets there is high agreement amongst the three nets, but none of them are very certain of their decision. All targets were from tumor or border regions. Compared to the 7 targets in Table 6 (the red zone of Fig. 5B) these NNs show more individual variation (higher *V*_*RA*_), less disagreement (lower *V*_*ER*_), and lower *P*_*Tumor*_ estimates. No NN configuration predicted tumor for all 17 targets. All three NN configurations agreed in their prediction of tumor for 6 samples (highlighted in bold). All three NN configurations also agreed when they predicted no tumor likely for 7 samples. In the remaining 4 samples at least one NN configuration predicted tumor, but probabilities were quite low ranging from *P*_*Tumor*_ ~ 0.505 to 0.634.

**Figure 7:**
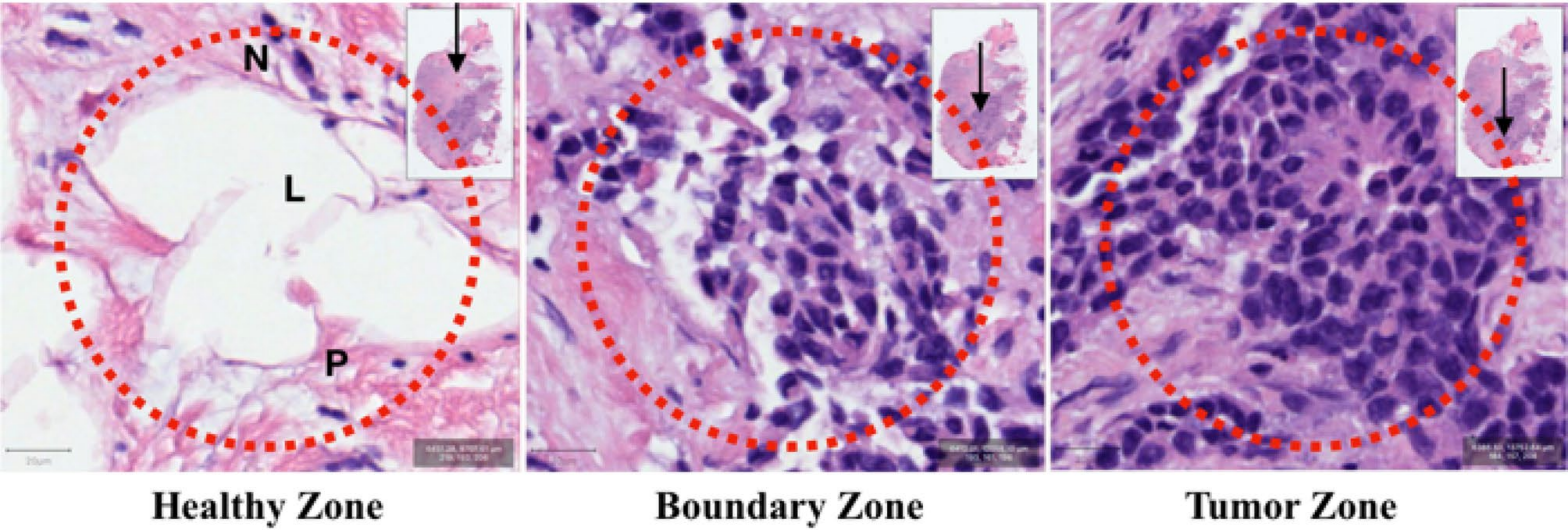
Tumor heterogeneity and laser spot size Laser spot size of 85 μm diameter (dotted red ring) superimposed on 100 × 100 μm regions from healthy, boundary, and tumor zones. Paraffin-filled white spaces (L) were lipid-rich in vivo. These healthy cells are surrounded by protein-rich supporting stroma (P). Cell nuclei (N) rich in DNA, RNA, and peri-nuclear proteins occur infrequently in healthy regions and increasingly dominate in boundary and tumor regions. Scale bar in lower left corner is 20 μm. Inset shows location (black arrow) of zones on the H&E slide.

Figure 7 shows a laser spot size of 85 μm diameter (the dotted red rings) superimposed on 100 × 100 μm regions from healthy, boundary, and tumor zones. The inset shows the location (black arrow) of the regions on the H&E slide. The scale bar in the lower left corner of each image denotes 20 μm. Paraffin-filled white spaces (L) are lipid-rich in vivo. In the first image, healthy cells are surrounded by protein-rich supporting stroma (P). Cell nuclei (N) rich in DNA, RNA, and peri-nuclear proteins occur infrequently in these healthy regions and then increasingly dominate in boundary and tumor zones. From the geometric constraints depicted in these images it appears that spectral mixtures of healthy and tumor signals can be expected to occur quite frequently when probing heterogenous, rapidly progressing tumors. Hence, probabilistic algorithmic outputs were chosen to represent the likely detection of a cluster of mixed cells.

**Figure 8:**
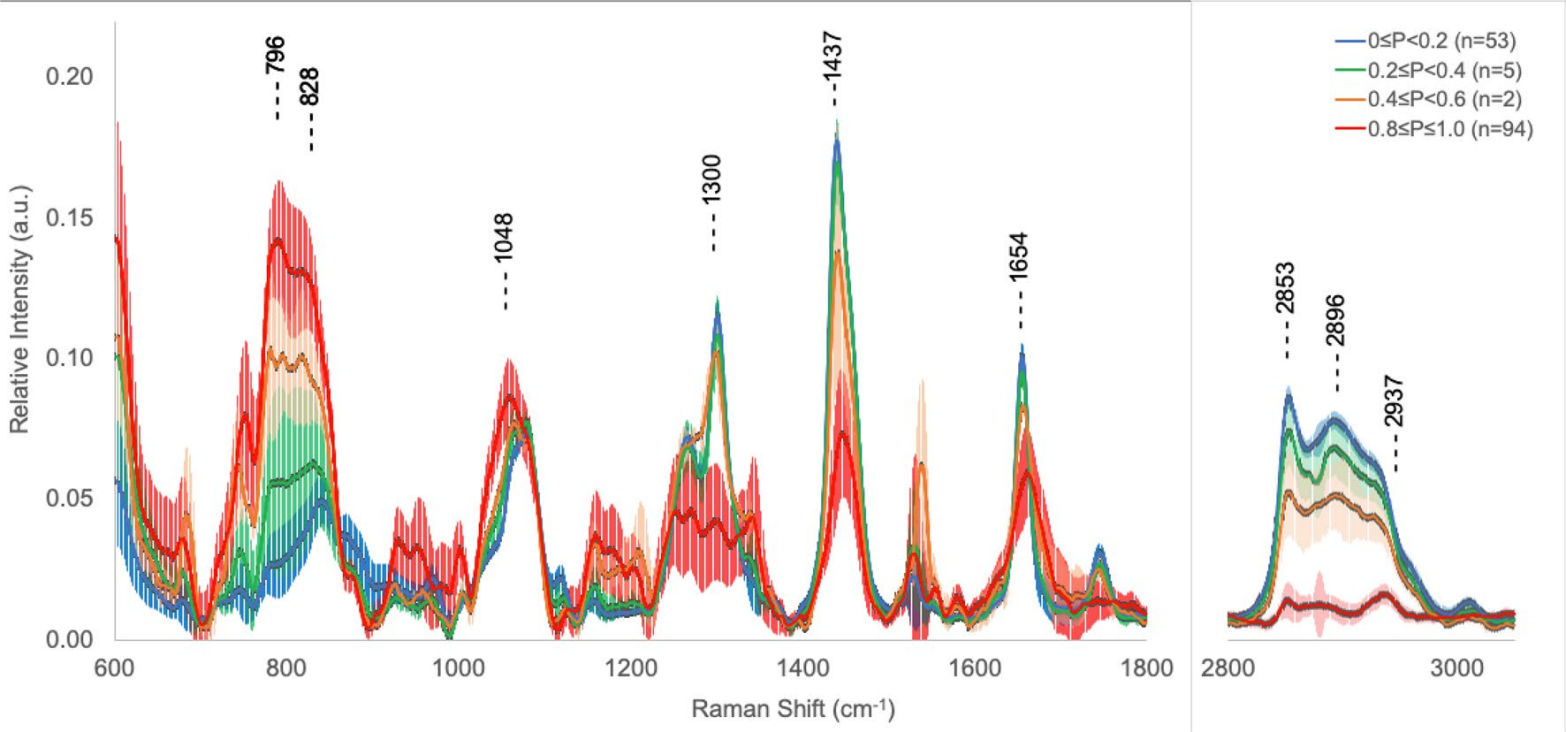
Spectra grouped by 5 equal bins of Bayesian probability generated by the k-means and NN autonomous classification with 1-sigma error bars (203 spectra, 8 patients). The maximum probability generated amongst the three networks (FP, HW, FPHW) were used to sort LRS targets into five (5) equally spaced probability bins: 0.0 ≤ *P* < 0.2; 0.2 ≤ *P* < 0.4; 0.4 ≤ *P* < 0.6; 0.6 ≤ *P* < 0.8 and 0.8 ≤ *P* ≤ 1.0. No spectra are classified in range 0.6 ≤ *P* < 0.8. Maximum signal separation occurs with bands for the DNA|RNA signal at 796 cm^−1^, the protein band at 828 cm^−1^, the lipid signal at 1437 cm^−1^ and the three HW bands. Distribution and cross-mapping of these probabilistic assignments with the original histopathological quintile assignment appears in Table 7.

The final statistics and spectra identified by the autonomous, probabilistic artificial intelligence techniques employed in this study are presented in Table 7. Table 7 shows the autonomous classification of the 154 spectra for which we have H&E quintile assignments. Here the spectra from the original five (5) histopathology quintiles have been re-binned according to the maximum probability predicted by any one the three NNs (FP, HW, FPHW) when classifying the full data set (n = 203, 8 patients). The Bayesian probability quintiles are equal range bins: 0.0 ≤ *P* < 0.2; 0.2 ≤ *P* < 0.4; 0.4 ≤ *P* < 0.6; 0.6 ≤ *P* < 0.8 and 0.8 ≤ *P* ≤ 1.0. The mean spectra corresponding to these quintiles appear in Fig. 8. 147 of 154 spectra (95.4%) were assigned to end member quintiles: 0.0 ≤ *P* < 0.2 (N = 53, tumor unlikely) and 0.8 ≤ *P* ≤ 1.0 (N = 94, tumor highly likely). No targets received a probability score in the 0.6 ≤ *P* < 0.8 range. 24 of 25 spectra (96%) classified by histopathology as healthy did not receive a cancer prediction > 0.2 from the autonomous classification. The one spectra in the 0.2 ≤ *P* < 0.4 bin received a maximum score of *P* = 0.21. For the 49 spectra obtained from regions deemed 100% tumor-rich by histopathology, 45 (91.8%) were placed in the highest probability quintile 0.8 ≤ *P* < 1.0 bin. Two spectra were considered highly likely to originate in healthy tissue (0.0 ≤ *P* < 0.2) and two others appeared in the 0.2 ≤ *P* < 0.4 bin. The average spectra for each of the other four probability quintiles appear in Fig. 8. Maximum signal separation occurs with bands for the DNA|RNA signal at 796 cm^−1^, the protein band at 828 cm^−1^, the lipid signal at 1437 cm^−1^ and the three HW bands.

**Table 7:** Autonomous network assignment of cancer likelihood.

Histopathology	Maximum NN probability of cancer quintiles					
Quintile	0.0 ≤ *P* < 0.2	0.2 ≤ *P* < 0.4	0.4 ≤ *P* < 0.6	0.6 ≤ *P* < 0.8	0.8 ≤ *P* ≤ 1.0	N
100%	2	2	0	0	45	49
75%	12	2	0	0	35	49
50%	6	0	1	0	12	19
25%	9	0	1	0	2	12
0%	24	1	0	0	0	25
N	53	5	2	0	94	154

Using the maximum prediction of cancer likelihood generated by NNs trained on FPHW, FP, or HW inputs with autonomous k-means classification as the gold standard, the original histopathology quintiles are cross-matched to five (5) equally spaced cancer probability bins. 147 of 154 spectra (95.4%) were assigned to end member quintiles: 0.0 ≤ *P* < 0.2 (N = 53, tumor unlikely) and 0.8 ≤ *P* ≤ 1.0 (N = 94, tumor highly likely). No targets received a probability score in the 0.6 ≤ *P* < 0.8 range. The average spectra for each of the other four probability quintiles appear in Fig. 8. 24 of 25 spectra (96%) classified by histopathology as healthy did not receive a cancer prediction > 0.2 from the autonomous classification. The one spectra in the 0.2 ≤ *P* < 0.4 bin received a maximum score of *P* = 0.21. For the 49 spectra obtained from regions deemed 100% tumor-rich by histopathology 45 (91.8%) were placed in the highest probability quintile 0.8 ≤ *P* < 1.0 bin. Two spectra were considered highly likely to originate in healthy tissue (0.0 ≤ *P* < 0.2) and two others appeared in the 0.2 ≤ *P* < 0.4 bin.

The data presented here indicate that a panel of unsupervised, autonomous, stochastic nonlinear neural networks trained on both broad and focused infrared laser Raman spectroscopy data, can provide an operating team with Bayesian probability estimates that a tissue region contains cancerous cells. The networks all learn from spectral bands rich in information from four of the major cellular constituents: DNA|RNA, protein, carbohydrate, and lipid. However, for 7 out of the 203 spectra evaluated (~ 3.4%), the NNs trained on full spectrum, fingerprint region, and high wavenumber bands showed significant variability in their estimation of cancer likelihood. For conservative, real time management of surgical patients, the availability of multiple estimates of the likelihood of tumor provides the surgical team with a broader safety net. The NNs trained on high wavenumber data appear particularly sensitive to loss of signals from the destruction of C–H bonds, a potential early warning sign of the chaotic disruption of cell structure. However, a “loss of signal” signature can leave the surgeon uncertain about whether this is a global defect in signal acquisition, or a true indicator of tissue damage. The appearance of a strong increase in signal intensity for bands attributable in DNA|RNA, and/or to protein detected in the fingerprint region of the spectra can provide critical information in determining the origin of the high wavenumber signal collapse. For several spectra the alteration in nucleotide and protein signals was the only warning signal that would have been available to the clinician.

## Discussion

There are two succinct takeaways from the data reported here. (1) There is a strong correlation between NNs trained by autonomous k-means spectral classification and by histopathology “gold standards”. (2) The detection of what may be subtle early signs of tumor by the HW NNs is noteworthy, but not unexpected. LRS high wavenumber measurements have previously been shown to be reliable markers of breast cancer cell evolution in immortalized, transformed, and invasive cells^31^, and can accurately estimate the loss of lipid content during in vivo evaluation of breast cancer progression^44^. Evaluation of our spectra revealed the decrease in HW flux warning of alterations in C–H bonds in DNA|RNA, protein, carbohydrates, and lipids. C–H bonds are one of the most abundant Raman targets in human tissue and one of the bonds the most easily altered during cellular growth, exposure to high energy radiation, or changes in pH, acidity, or hydration. The subtle changes in C–H signatures detected by HW can apparently be masked in the FPHW analysis by strong lipid signals in the fingerprint region.

It should be noted that all three NNs show a significant range of probabilities for spectra obtained from both healthy and tumor-rich regions. It should not be surprising that mixed signatures occur when probing aggressive tumors in vivo. The laser probes commonly used for LRS acquire their data from a tissue volume approximately 100 μm in diameter. Breast cancer cells observed during histological evaluation in this study range in diameter from 10 to 20 μm and are often interspersed with relatively healthy collagenous and lipid tissues, particularly in boundary conditions.

In this communication, we report that autonomous machine algorithms can mine the information content of infrared Raman spectra to distinguish healthy from cancer-containing breast tissue. The evaluation can be accomplished on a time scale of minutes instead of the days to weeks required at present for histopathological evaluation. We identify nine spectral regions useful in comparing DNA|RNA, protein, carbohydrate, and lipid cellular components of healthy and cancer cells. Six of these bands are in the Raman spectrum fingerprint region (FP) (600–1800 cm^−1^) and three are in the high wavenumber (HW) region (2800–3000 cm^−1^). Three stochastic nonlinear NNs were trained on either FP, HW, or FPHW bands to estimate the Bayesian probability that a spectrum exhibits changes expected in cancer tissue. To demonstrate the possibility of replacing a two-week wait for histopathology with a real-time tumor detection using only spectra, training was accomplished with two gold standards. In a first experiment, the three NNs used histopathology diagnostics as their gold standard for training. In the second experiment, the autonomous classification of the spectra by k-means produced the gold standard. In both experiments one of the three NNs provided a broadband analysis of the spectral data using all nine bands. The two other networks focused on data from only the FP (6 bands) or HW (3 bands) regions. Our data indicate that loss of signal in HW bands can serve as an early warning marker of tissue destruction, while the relative intensity of two strong FP bands may be particularly sensitive to the shifts in RNA, DNA, and protein composition characteristic of proliferating breast cancer. Finally, we demonstrate that even though ~ 96% of the time any of the three NNs can distinguish between healthy and tumor tissue, for 7 of 203 spectra only the availability of data from all three would have ensured a detection of tumor activity. Our data indicate that without the multi-network approach described here, critical early diagnostic signs may be hidden in analyses that rely only on full spectrum data or on data from only FP or HW regions. The ability to (1) poll the disparate diagnostic strengths of multiple algorithms and (2) assess stochastic NNs prediction certainty, adds a needed level of transparency to the interaction between machine algorithm and practicing surgeon. It also provides a way forward for laboratory experiments designed to identify the fundamental biomolecular shifts occurring in tumor evolution.

There are limitations when testing this technique. An H&E stained tissue sample is a micrometer scale 2-D sampling (a 4 μm thick tissue slice) of a centimeter scale 3-D resected tissue. In the protocol for this study pathology laboratory personnel are not asked to deviate from standard clinical evaluation. 2-D orientation of the sample is maintained, but z-axis depth for extraction of the slide material is not constrained. The infrared LRS probe samples a site approximately 0.5 to 1.0 mm deep into the tissue, a depth that can be slightly above or below the H&E stained tissue (most likely above). Solving the disconnect between the 3-D and 2-D technologies will require modification of clinical protocols to significantly increase H&E sampling along the z-axis. This technical issue is beyond the scope of the current study and does not affect the autonomous machine language diagnostic technique central to these experiments. This study utilizes tissue biomarkers from regions that are visible to the surgeon and used in the post-operative pathology assessment, therefore this study looks at information from tissue that is used in the current accepted standard of care. Machine learning algorithms employed over the last decade in LRS breast cancer investigations have often not provided two critical pieces of information important to the practicing surgeon: a probability that a classification is correct and the expected error in that probability. Stochastic backpropagation artificial neural networks inherently provide both pieces of information not simply for clusters of data, but specifically for each tissue site examined by LRS.

## Methods

### Raman instrumentation

The Raman instrumentation has been described in a previous communication^43^. All experiments employed B&W Tek’s 785 nm system, the i-Raman Plus. The i-Raman Plus uses a high quantum efficiency 2048-pixel CCD array detector with a spectral resolution of 4.5 cm^−1^ and a spectral coverage range from 147–3350 cm^−1^. The detector cooled temperature is  − 2 °C with a typical dynamic range of 50,000:1. The effective pixel size is 14 μm × 9 μm. The integration time and exposure time is 30 s (single accumulation) for each sample and the laser power at sample is 100mW.

The system is highly portable. The spectrometer housing connects via fiber optic cables to the BAC102 Raman Trigger Probe. The probe has a spot size of 50–85 μm. The BAC150B probe holder can be used to stabilize the probe for benchtop data collection, or the probe can be handheld during use in the surgical field. Alternatively, the probe can be integrated with B&W Tek’s BAC151B video sampling system, or the BAC104 adapter can be used to integrate the probe with a standard laboratory Olympus microscope. All data discussed in this article were taken using the standard BAC150B probe.

### Tissue preparation and histology

Tissue samples were collected following surgical resection under Institutional Review Board (IRB) protocol (#16317) at City of Hope in Duarte, California (VJ, LL, YF). All experimental protocols used in this study were approved by City of Hope IRB. All methods were carried out in accordance with relevant guidelines and regulations. Informed consent was obtained from all patients.

Following resection, samples were immediately frozen and stored at  − 80 °C. For Raman spectral analysis, samples were thawed ~ 5–10 min before data collection. There are no histopathology or spectral alterations indicative of freeze artifacts. Once spectral data were obtained, the sample was formalin fixed and paraffin embedded, and standard H&E-stained slides were prepared. These slides were digitally scanned at 20X resolution using a Ventana iScan HT whole slide scanner (Roche Holding AG, Basel, Switzerland) and the resulting whole slide images were viewed using QuPath open source imaging software (Queen’s University Belfast, Belfast Northern Ireland, UK). Areas from which spectra were obtained were correlated with the H&E findings.

There was one tissue sample per patient and spectra were systematically taken from all regions of the tissue sample. Histopathology was obtained for all tissue samples. A subset of 154 regions (1 mm^2^) of H&E tissue stains centered around laser target site were interrogated using QuPath image processing software by expert pathologist to generate a quintile assessment of cancer involvement (0%, 25%, 50%, 75% and 100% tumor).

### Spectral preprocessing

The raw spectral data files were extracted from the B&W Tek’s software. Standard preprocessing methods for Raman spectral data were accomplished in MATLAB 2017b^45,46^ as follows:

- The region between 147–600 cm^−1^ was removed since it exhibits fluorescence from the fiber optic feedback loop and bleedthrough from the laser.
- Fluorescence correction was implemented by calculating a baseline for the spectra by using an asymmetrically reweighted penalized least squares smoothing algorithm, arPLS, developed by Baek and coworkers^47^. This method utilizes an iterative process to determine the noise and correspondingly updates the weights in order to calculate a baseline. This calculated baseline was subtracted from the intensity values of the raw spectrum in order to perform a fluorescence removal. The arPLS parameters are lambda = 10^5^ and ratio = 10^–3^.
- Spectra were smoothed by Savitzky-Golay algorithm with a sliding window encompassing 7 bands^48^.
- Each spectrum was centered around its’ mean by subtracting the mean of the spectrum from every data point in the spectrum.
- Finally, each spectrum was divided by its Euclidean norm in order to normalize each spectrum to a vector of length 1.

For publication plotting purposes, we have removed the dead zone between 1800 and 2800 cm^−1^ for all figures.

### Neural networks to generate bayesian estimate of cancer

Bayesian probability theory presents a formalized methodology for establishing the likelihood that any particular observation can be correctly included in a specific class of event^49^.

Bayesian theory is a fundamental tool used to quantify how human experts generate reproducible classification decisions. Machine learning algorithms known as neural networks (NNs), have become a widely used tool for turning Bayesian theory into practical application. NNs were originally implemented as simple optimization algorithms modeled on signal processing characteristics of the human brain^50–54^. NNs compress data and extract discriminatory features from a data set in a manner similar to PCA. NNs can also model non-linear interactions and distinguish classes that are not linearly separable, feats beyond the capabilities of PCA. However, the most powerful feature of stochastic nonlinear NNs is their ability to not only provide target classification, but also generate a Bayesian probability estimate of the correctness of their decision for each individual target. NNs constructed as stochastic back propagation algorithms were predicted theoretically^55,56^, and then shown experimentally to be^57^, robust Bayesian estimators.

Neural network architecture and computational methods used in this study have been described previously^58–61^. All networks in the experiments reported here are constructed and run in MATLAB 2017b. It is important to realize that the NN will provide a probability of class inclusion for all proposed classes. Additionally, if the human expert classification is in error, NNs prove remarkably adept at disagreeing, i.e. they will provide a data-driven, best classification estimate even if the ‘gold standard’ diagnosis is incorrect. For a formal review of Bayesian probability theory and neural network estimation of Bayesian probabilities, see Supplementary material.

### Comparing gold standards: training NNs using histopathology versus autonomous k-means spectral classification

During training of a stochastic backpropagation algorithm an output “gold standard” must be provided. For the first experiment we trained NNs using first histopathology classification and then autonomous k-means classification of Raman spectra as the gold standard. In the first case, NN output node values are compared to the expert opinion—in this study, the pathologist's evaluation of standard H&E stained post-operative slides. The pathologist (DS) examined 154 images of tissue in a 1 mm^2^ area centered around the target site for the Raman probe. The fraction of tissue deemed to contain at least some cancer was recorded as 0, 25, 50, 75, or 100%. A total of 74 targets were determined to be either completely devoid of tumor (T = 0%, n = 25) or entirely involved by tumor (T = 100%, n = 49).

For NN training and evaluation this data subset of 74 targets were assigned output vectors of [1,0] for tumor sites, [0,1] for apparently healthy regions according to, first, the histopathological diagnostic and, second, according to the autonomous classification by k-means.

Since it is not possible to retrieve histological data for each spectral sample, unsupervised autonomous algorithms are explored in this study. For autonomous classification we replace the tentative class membership assignment derived from histopathology with direct unsupervised examination of each Raman spectrum using a machine learning clustering algorithm, k-means^62^. The k-means algorithm simply starts with k groups, each consisting of a single random point, and then adds each new point to the group with the mean nearest to the location of the new point. After a point is added to a group, the mean of that group is recalculated to incorporate the new point. At each step the k-means are, in fact, the means of the groups they represent, hence the algorithm is known as k-means. In this study, the k-means classification was implemented in MATLAB using the squared Euclidean distance metric and the k-means ++ algorithm for cluster center initialization^45^. Once the algorithm has tentatively assigned each spectrum to a cluster, stochastic backpropagation NNs then generate the Bayesian probability that the k-means clustering has successfully identified the appropriate class just as they did when using the histopathology diagnostic as the gold standard.

To test an entire data set, a leave-one-out round-robin procedure using multiple nets is employed in all experiments. In this strategy all the data are used for training except for one spectrum. That spectrum is then used as a test sample for the trained network. The training and testing are repeated cycling through all members of the data set until all spectra have been classified by networks that have not seen the test spectrum during training.

We implemented this technique by modifying the MATLAB 2017b Neural Network Pattern Recognition toolbox with 9, 6, or 3 input nodes, 3 nodes in second layer and 2 output layer nodes for NNs FPHW, FP, and HW, respectively. The implementation employed the scaled conjugate backpropagation algorithm and Softmax transfer function for the output layer. Following training and testing of the six stochastic backpropagation NNs to compare the histopathology and autonomous gold standards, the autonomous classification using k-means and NNs was extended to the full spectral data set (n = 203, 8 patients).

## Supplementary Information

Supplementary Information
