# Supplementary material for: Raman spectroscopy and artificial intelligence to predict the Bayesian probability of breast cancer
Source: Sci Rep. 2021 Mar 22;11:6482. doi: 10.1038/s41598-021-85758-6 (PMC7985361; doi:10.1038/s41598-021-85758-6)

**Raman Spectroscopy and Artificial Intelligence to Predict the**

**Bayesian Probability of Breast Cancer**

Ragini Kothari ^1,2^, Veronica Jones^1^, Dominique Mena^2^, Viviana Bermúdez Reyes^2^, Youkang Shon^2^, Jennifer P. Smith^3^, Daniel Schmolze^4^, Philip D. Cha^2^, Lily Lai^1^, Yuman Fong^1^, Michael C. Storrie-Lombardi^3,5^

*1. Department of Surgery, City of Hope, 1500 E. Duarte Rd., Duarte, California 91010*

*2. Department of Engineering, Harvey Mudd College, 301 Platt Blvd., Claremont, California 91711*

*3. Department of Physics, Harvey Mudd College, 301 Platt Blvd., Claremont, California 91711*

*4. Department of Pathology, City of Hope, 1500 E. Duarte Rd., Duarte, California 91010*

*5. Kinohi Institute, Inc., Santa Barbara, California 93109*

**Supplementary Material**

**Comparison of Heuristic Feature Selection with Global PCA**

In a previous communication, PCA performed on the entire spectral bandwidth along with LDA was shown to distinguish between cancerous and healthy spectra. However, this paper employs a preliminary feature selection method in order to provide increased transparency on what features contribute to the output of the pattern recognition NN while still minimizing (and optimizing) the number of variables used by the clustering and classification algorithms, a goal identical to our earlier use of PCA. Unfortunately, blind PCA extraction of data from the entire spectral bandwidth can be compromised by random variables unrelated to pathophysiology such as surgical dye marking during tumor excisions. These bands are spread across both the FP and HW regions. To make our technique as reproducible as feasible without inserting experimental bias we have relied on identifying the spectral regions of maximum variance (information). However, since we then specifically selected data from high information spectral regions exhibiting no overlap in flux distributions from ink stains, the “human intervention” in the variable selection process makes it mandatory that we refer to our data selection process as “heuristic”. Unsupervised k-means classification is performed on the dataset post feature selection with both heuristics and PCA. Table S1 displays these results as a confusion matrix with the k-means on the PCA dataset as the gold standard. Here we see that the heuristic feature section produces extremely comparable results to the PCA, and thus the heuristic feature selection used for our results ensures no information loss.

**Table S1. Confusion Matrix Comparing K-means classification with PCA and Heuristic Feature Selection (n= 203)**. K-means classification is almost identical after both PCA and heuristic feature selection, they differ in the classification of one data point, identified as tumor after PCA and as healthy after heuristic feature selection by k-means.

|  | Heuristic Tumor | Heuristic Healthy | Total |
| --- | --- | --- | --- |
| PCA Tumor | 117 | 1 | 118 |
| PCA Healthy | 0 | 85 | 85 |
|  |  |  | 203 |

**Neural Network Analysis Using Either H&E or k-means as Gold Standard with Balanced Classes.**

Figure S1 compares the probabilities generated by the neural network trained on k-means to the probabilities generated by the neural network trained on histopathological classification of spectra likely to be either 100% or 0% tumor with balanced classes (i.e. n = 25 for both healthy and tumor classes). 25 spectra from the tumor class (originally n = 49) were randomly chosen to create the balanced classes. Figure S1 exhibits similar data distribution as compared to Figure 3 (which contains unbalanced classes).

**Figure S1. Bayesian probabilistic classification for 50 spectra from 5 patients using either H&E staining (x-axis) or k-means unsupervised spectral clustering (y-axis) as training gold standard.** Spectra were from regions classified by histopathology as likely to be composed of either 100% or 0% tumor. Networks were trained using as inputs (**x**) 6 bands from the FP region (**FP**); (△) 3 bands from the HW region (**HW**); or (●) the full set of 9 bands (**FPHW**). Targets obtained from tissue in tumor-rich regions according to H&E stain are denoted by **red** markers**,** while data obtained from areas apparently devoid of tumor are denoted by **blue** markers.


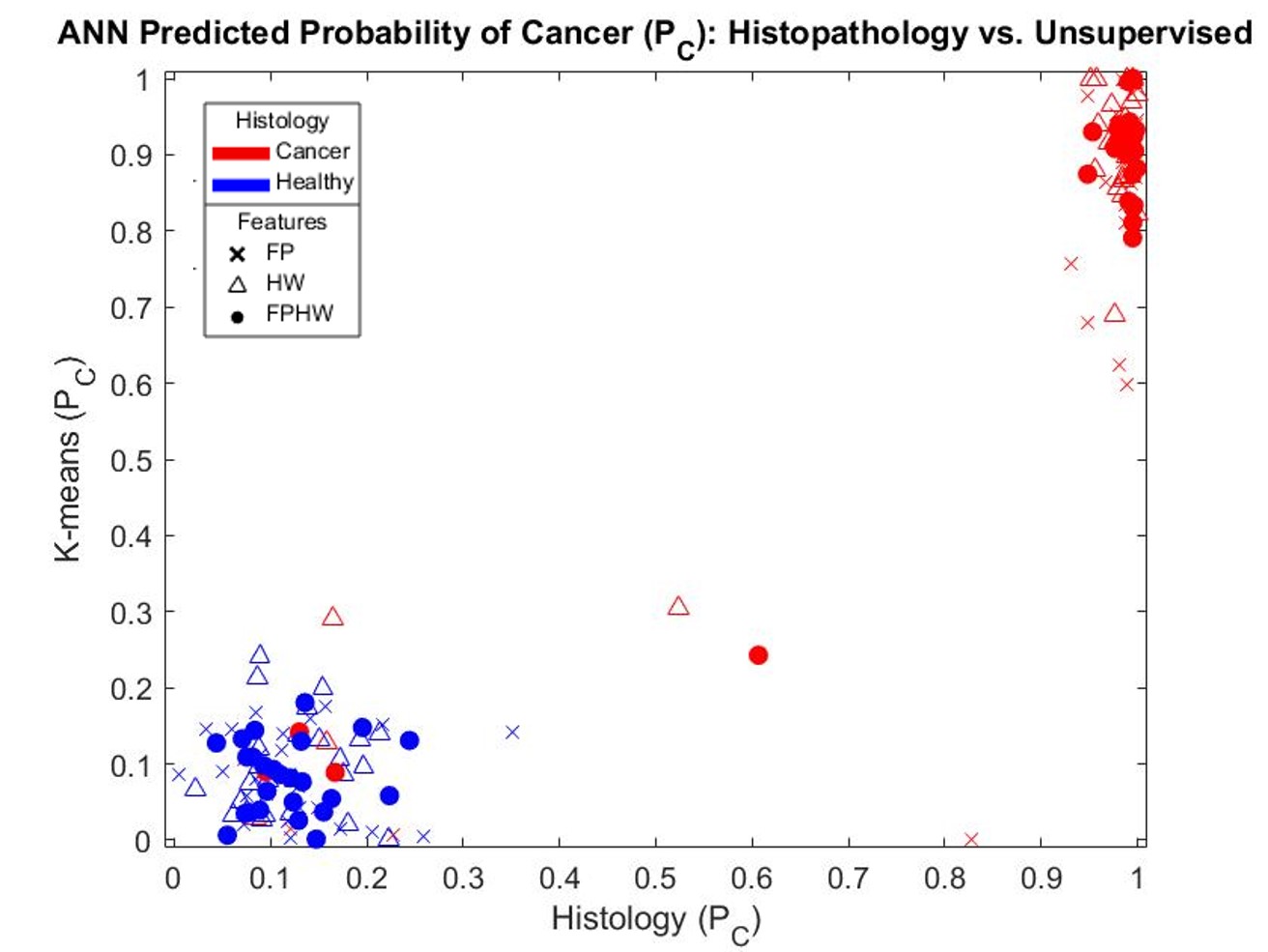


**Bayesian Probability Theory**

In brief, propose a hypothesis, *H*, that predicts an observation, *X*, of unknown class membership, belongs to a specific class, *C*. For classification, determine *P(H|X*), i.e., the probability, *P*, that the hypothesis, *H*, holds given the observation, *X*. *P(H|X)* is the *a posteriori* probability of *H* given *X*. In contrast, *P(H)* is the *a priori* probability of *H*, or the likelihood of including any observation, *X*, in class *C*. The posterior probability, *P(H|X)*, is based on more information (such as the occurrence of *X*) than the prior probability, *P(H)*, which is independent of *X*. Bayes theorem provides a way of calculating the *a posteriori* probability, *P(H|X)*, from *P(H)*, *P(X)*, and *P(H|X)*. Bayes theorem is

*P(H|X) = P(X/H) P(H) / P(X)*

Therefore, the probability of H given X is directly proportional to the product of the probability of X given H and the probability of H, and is inversely proportional to the probability of X.

**Artificial Neural Networks to Estimate Bayesian Probabilities**

In brief, back propagation NNs are built in layers of nodes that include an input layer, at least one middle or 'hidden' layer, and one output layer. Experimental data are provided to the input layer. Each node in the input layer feeds data forward to the next layer with data values multiplied by a weighting function that will be modified during network training. Each node in the hidden layer then sums all the weighted input data and feeds that sum forward to the next layer. A node at layer ***s*** calculates a linear combination over the input elements *x_j_^(s-1)^* from the previous layer according to

*I_i_^s^ = ∑_j_ w_ij_^s^ x_j_^(s-1)^*

where the *w_ij_* ’s are the weights associated with each connection between nodes. Where *i* refers to the components of the output and *j* refers to the components of the input. Each node then fires off a signal *x^s^ = f(z)* according to a non-linear threshold function usually of the form

*f(z) = 1/[1+exp(z)]* (in the interval [0,1]),

where z = *I^s^*. Once the architecture of an NN is determined, the weights, *w_ij_*, (the free parameters) are initialized with random starting values and then determined by minimizing ‘least-squares’ cost using a simple chain rule (gradient descent). For each sample in a training set of data, the NN compares its output vector in a classification space ***o*** to the desired vector ***d*** provided by a human expert. The expert can provide as many classes as needed, but all elements of each vector are zero except for the one element set to1 corresponding the classification predicted by the expert. The comparison of the two vectors is accomplished in terms of a cost function usually of form

E = ½ *= ∑_k_ (****o****_k_ -* ***d****_k_ )^2^*

where the sum is performed over the components of the vectors. This cost function is minimized with respect to the free parameters, *w_ij_*. The NN weights are then reverse updated starting with the output layer and moving back across each successive layer implementing a small change in each time step (t),

*Δ w_ij_ (t+1) = -* ***n*** *∂E/∂ w_ij_ +* ***α****Δ w_ij_ (t)*

where the rate of learning can be modulated using the “learning coefficient”, ***n***, and the “momentum”, ***α***. In our case alpha, *α****,*** was set to 0. Once training is complete, the NN is ready to classify targets not previously encountered during training. The *jth* component of the output vector is the NN estimate of the Bayesian probability for inclusion in class *j* given the input parameters *P(Cj|x)*. The Bayesian nature of the output and an evaluation of the training accuracy of an NN can be assessed by checking that the sum of the NN output vector components is *∑_k_* ***o****_k_* ~ 1.

**Table S2. NN predictions exhibiting low inter-NN variance (*V_ER_* < 1σ) and high intra-NN variance (*V_RA_* > 1σ)**. These data are for the 17 targets found in the yellow region of Figure 5. All targets were from tumor or border regions. Compared to the 7 targets in Table 6 (the red zone of Figure 5) these NNs show more individual variation (higher ***V_RA_***), less disagreement (lower ***V_ER_***), and lower probabilities (*P_Tumor_*) that a spectrum originated in tumor tissue. No NN configuration predicted tumor for all 17 targets. All three NN configurations predicted tumor for 6 samples (highlighted in **bold**). All three NN configurations predicted no tumor for 7 samples (highlighted in ***bold italic***). In the remaining 4 samples at least one NN configuration predicted tumor, but probabilities were quite low ranging from *P_Tumor_* ~ 0.505 to 0.634.

|  |  |  | Intra-NN Variance (V_RA_) | | | Tumor Probability (*P_Tumor_*) | | |
| --- | --- | --- | --- | --- | --- | --- | --- | --- |
| Histology | Region | V_ER_ | FP | HW | FPHW | FP | HW | FPHW |
| - | T | 0.013 | 0.047 | 0.058 | 0.053 | 0.602 | 0.379 | 0.444 |
| 50 | B | 0.013 | 0.062 | 0.046 | 0.035 | 0.408 | 0.508 | 0.634 |
| 100 | T | 0.010 | 0.005 | 0.056 | 0.005 | ***0.130*** | ***0.323*** | ***0.189*** |
| - | T | 0.009 | 0.004 | 0.072 | 0.007 | **0.895** | **0.729** | **0.890** |
| - | T | 0.007 | 0.069 | 0.056 | 0.098 | 0.463 | 0.631 | 0.541 |
| - | T | 0.006 | 0.070 | 0.026 | 0.024 | ***0.360*** | ***0.248*** | ***0.210*** |
| - | T | 0.005 | 0.006 | 0.024 | 0.002 | **0.879** | **0.791** | **0.932** |
| - | B | 0.005 | 0.009 | 0.020 | 0.012 | ***0.127*** | ***0.263*** | ***0.164*** |
| 75 | B | 0.004 | 0.035 | 0.018 | 0.004 | ***0.216*** | ***0.219*** | ***0.102*** |
| 100 | T | 0.004 | 0.057 | 0.002 | 0.006 | **0.796** | **0.924** | **0.890** |
| 25 | B | 0.003 | 0.006 | 0.018 | 0.028 | ***0.111*** | ***0.182*** | ***0.222*** |
| - | T | 0.003 | 0.049 | 0.003 | 0.005 | **0.819** | **0.922** | **0.908** |
| 25 | B | 0.002 | 0.026 | 0.002 | 0.003 | **0.857** | **0.923** | **0.933** |
| - | T | 0.001 | 0.070 | 0.085 | 0.031 | 0.497 | 0.505 | 0.438 |
| 50 | T | 0.001 | 0.032 | 0.065 | 0.066 | **0.768** | **0.707** | **0.722** |
| - | T | 0.000 | 0.012 | 0.009 | 0.021 | ***0.137*** | ***0.122*** | ***0.163*** |
| - | B | 0.000 | 0.005 | 0.049 | 0.013 | ***0.210*** | ***0.209*** | ***0.188*** |
| **^1^**Pathologist estimate of likelihood probe would strike tumor tissue in 1 mm^2^ region around laser.  **^2^**Macroscopic (1X) visual assessment of spectra collection sites as tumor (T), healthy (H), or boundary (B) regions. | | | | | | | | |

**Raman Spectra of Commonly Used Surgical Inks**

Figure S2 depicts the Raman spectra of commonly used surgical inks as well as average healthy and tumor spectra of breast tissue (not florescence corrected). Feature selection strategically avoided prominent ink peaks.

**Figure S2**. Raman spectra of surgical inks.


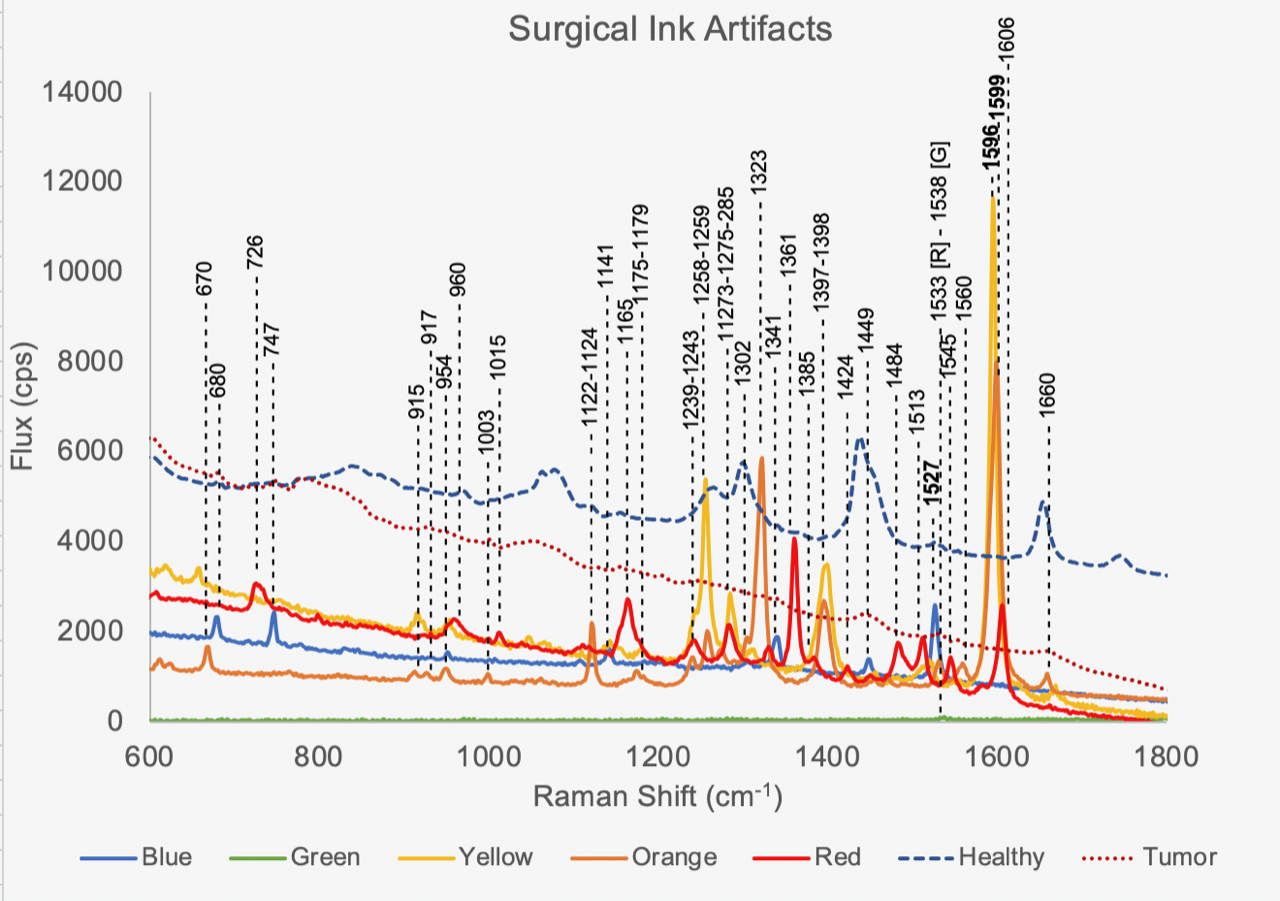

Supplement: Supplementary file 1 — Supplementary Information [file 41598_2021_85758_MOESM1_ESM.docx]
